# Excess mortality and hospitalizations in transitional-age youths with a long-term disease: A national population-based cohort study

**DOI:** 10.1371/journal.pone.0193729

**Published:** 2018-03-13

**Authors:** Antoine Rachas, Philippe Tuppin, Laurence Meyer, Bruno Falissard, Albert Faye, Nizar Mahlaoui, Elise de La Rochebrochard, Marie Frank, Pierre Durieux, Josiane Warszawski

**Affiliations:** 1 Center for Research in Epidemiology and Population Health UMR 1018, INSERM, Paris-Sud University, Versailles Saint-Quentin-en-Yvelines University, Villejuif, France; 2 Department of Epidemiology and Public Health, Paris Sud University Hospital, Assistance Publique-Hôpitaux de Paris, Paris, France; 3 Paris Sud University, Le Kremlin-Bicêtre, France; 4 Caisse Nationale d'Assurance Maladie des Travailleurs Salariés, Paris, France; 5 Paris 7 Denis Diderot University, Sorbonne Paris Cité, Paris, France; 6 General Pediatrics Unit, Robert Debré Hospital, Assistance Publique-Hôpitaux de Paris, Paris, France; 7 INSERM U1123, Paris, France; 8 French National Reference Center for Primary Immune Deficiencies (CEREDIH), Necker Enfants Malades University Hospital, Assistance Publique-Hôpitaux de Paris, Paris, France; 9 Pediatric Immuno-Hematology and Rheumatology Unit, Necker Enfants Malades University Hospital, Assistance Publique-Hôpitaux de Paris, Paris, France; 10 INSERM UMR 1163, Laboratory of Human Genetics of Infectious Diseases, Necker Branch, Paris, France; 11 Paris Descartes University, Sorbonne Paris Cité, Paris, France; 12 Institut National d’Etudes Démographiques (INED), Paris, France; 13 Department of Medical Information, Paris Sud University Hospital, Assistance Publique-Hôpitaux de Paris, Paris, France; 14 Department of Informatics and Public Health, Georges Pompidou European Hospital, Paris, France; 15 INSERM Cordeliers Research Center UMRS 872, Paris Descartes University, Paris, France; Universidade Nova de Lisboa Instituto de Higiene e Medicina Tropical, PORTUGAL

## Abstract

**Introduction:**

The number of adolescents with a severe chronic disease has increased in high-income countries due to improvements in the prognosis of childhood-onset chronic conditions. The transition from childhood to adulthood is a critical period that may be associated with increased mortality and morbidity. We aimed to estimate the prevalence of adolescents with a long-term disease (LTD) in France and assess their mortality and hospitalization risks relative to the general population.

**Materials and methods:**

We extracted a population-based cohort from the French national health insurance database that included 61,119 subjects who reached 14 years of age between 2005 and 2014. LTDs are diagnosed by patients’ physicians and then confirmed and registered by a physician of the national health insurance system. We assessed mortality and hospitalizations using data of patients who were between 14 and 21 years-old.

**Results:**

Among 14-year-old adolescents, 3.30% (95% confidence interval: 3.16–3.44) had a LTD. Their mortality rate between the ages of 14 and 21 years was 20.9/10,000 person-years (13.7–32.1) *versus* 1.9 (1.5–2.5) for adolescents without a LTD. Mortality was higher in males than females in youths without a LTD, but not in those with a LTD. We found a similar pattern for the risk of hospitalization for an external cause. The five-year probability of hospitalization was 61.8% among youths with a LTD *versus* 42.7% for those without. The rate of planned hospitalizations sharply fell at 19 years-of-age among youths with a LTD, whereas the rate of unplanned hospitalizations remained stable.

**Conclusion:**

The 3% of youths with a LTD have ten-fold higher mortality than those without and a high risk of hospitalization. The decrease in the rate of planned hospitalizations at age 19 among youths with a LTD may indicate differences in medical practice after transfer to adult care or a break in medical care.

## Introduction

The prognosis of childhood-onset chronic conditions has improved in recent decades in high-income countries, such that more patients now reach adulthood. For example, the proportion of cystic fibrosis patients reaching adulthood (>18 years of age) increased from 27% to 56% between 1982 and 2007 and is forecasted to reach approximately 75% by 2025 in Western European countries [1,2]. It is estimated that almost 90% of children with congenital heart diseases will survive into adulthood, together with 94% of children with sickle cell anemia [3,4]. The population of perinatally HIV-infected patients is also ageing [5]. Better survival has likely led to an increase in the number of adolescents living with a chronic disease, but little national-level data are available on this subject.

Youths living with a chronic disease since childhood share many concerns during transition to adulthood [6], including taking increasing responsibility for their health and healthcare [7–10]. It is important to study this period of life, because adolescence is a critical period that may be associated with poor outcomes [6]. Epidemiological data on mortality and healthcare use in adolescents and young adults are yet sparse and often limited to a specific disease. Some studies have reported higher rates of healthcare use, either planned, unplanned, or both after entry into adolescence or young adulthood for patients with a childhood-onset chronic disease [11–17]. Conducting non-disease-specific studies could provide researchers and decision makers with original insights on how transition to adulthood is related to prognosis and planned or unplanned healthcare use. Such information could lead to a better understanding of the epidemiology of these diseases in adolescents and young adults and improvements in healthcare organization.

Furthermore, these youths must face the challenges of this period related to physiological, psychological, and socio-economic changes, as all adolescents. Conducting studies comparing youths with or without a chronic disease could help identify the role of the chronic condition in healthcare use versus that of entry into adulthood.

The French national health insurance database is one of the largest databases in the world and has been extensively used to guide public health policies in France, as these data allow systematic follow-up of all medical care received in France, including that of the low-income population [18–20]. With the Universal Health Coverage Act (opening up the right to statutory health insurance coverage on the basis of residence in France), data on almost all French residents, *i*.*e*., people living in France (with or without French nationality), are registered in this database. In addition, a list of major groups of chronic diseases, for which the severity and/or chronic nature requires prolonged treatment and costly therapy, has been established by decree as “*Affections de Longue Durée”* (long-term diseases, LTD) to allow patients to receive full reimbursement for expenditures related to the LTD. This provided the opportunity to estimate the prevalence of adolescents with a LTD diagnosed before 14 years of age at the national level and to describe the mortality and short-stay hospitalization rates between youths from 14 to 21 years of age, with or without a LTD.

## Materials and methods

### The EGB

The French national health insurance offers access to a large ongoing random sampling of 801,047 (at the time of this study) for research purposes, the *Echantillon Généraliste des Bénéficiaires* (EGB)[General Beneficiaries Sample] [18,21,22]. The EGB includes exhaustive records of hospitalizations in short-stay units (medical, surgical, obstetrical) from the national hospital discharge database, from 2005 to the end of 2015, and all data on LTD and mortality for subjects insured under the French general health insurance schemes, representing 80% of the population. The other types of hospitalizations (i.e., hospitalizations in psychiatric units, home-hospitalizations, and hospitalization in long-stay units) were not exhaustively recorded in the database during the study period and were thus not analyzed.

### Inclusion criteria

We selected all 61,119 adolescents from the EGB insured under the general scheme, who had reached the age of 14 years during the period from 01/03/2005 to 31/12/2014. We did not consider those who reached 14 after 2014 to allow at least 1 year of observation. This represents a sample of the generation born between 01/03/1991 and 31/12/1999 who were still alive and living in France at the age of 14.

### Study design

We selected a retrospective cohort using data of youths between the ages of 14 and 21 years to compare mortality and hospitalization rates between adolescents with or without a LTD. This age range, from one year before healthcare adulthood (in France, patients can be managed in adult care from the age of 15 years) to three years after legal adulthood (18 years old), thus covered the period of transition to young adulthood.

### Definition and classification of long-term diseases

LTD registration is obtained at the request of a patient’s physician and must be validated by the health insurance system physician, who codes the diagnosis using the International Classification of Diseases version 10 (ICD-10). Thirty-eight diseases registered before the age of 14 in our study population were identified as long-term, based on the LTD diagnosis codes. Trauma and burns were not considered as LTDs, nor tuberculosis, because such a condition should be resolved with antibiotic treatment and not last until adulthood. LTDs were classified into three categories defined in a previous Canadian study exploring youths with chronic health conditions [15]: chronic mental-health conditions, non-complex chronic conditions (chronic conditions typically affecting a single organ system), and complex chronic conditions (chronic medical conditions in which multiple morbidities and/or multi-organ manifestations are common) (S1 Table).

Overall, 2,019 subjects had at least one LTD registration before the age of 14 years, and were thus included in the LTD group for this study. The comparison group (N = 59,100) comprised the subjects without a LTD registration before the age of 14 years. Among them, the follow-up of those registered for a LTD after 14 (N = 879) was censored at LTD registration.

### Outcomes

We studied two types of outcomes: 1) all-cause mortality, using data from the national death registry and 2) hospitalizations in short-stay units. Two categories of hospitalizations were considered: unplanned, defined as an admission through an emergency department (ED) and planned, without admission through an ED. We also explored hospitalizations for an external cause, as a proxy of exposure to high-risk situations, such as injuries and road accidents, which are major causes of death in youths. An external cause was defined as a main diagnosis from the ICD-10 chapter “Injury, poisoning, and certain other consequences of external causes”, excluding complications of medical or surgical care, allergies, or intoxication by non-psychotropic drugs, which may be related to the LTD.

### Statistical methods

We first estimated the overall and gender-specific prevalence of the subjects with a LTD among adolescents aged 14 years. Then, overall and gender-specific annual mortality rates between the ages of 14 and 21 years were estimated by LTD status. Mortality rates were compared between females and males by estimating rate ratios with a 95% confidence interval (95%CI). The interaction between gender and LTD status towards mortality was tested using a Cox model. We then estimated the probability of being hospitalized by the age of 21 years, using the Kaplan-Meier method, by setting the time scale to age, and performed log-rank tests to compare survival curves. The repartition of the main diagnosis (ICD-10 chapter) of each stay was described by LTD status and gender for planned and unplanned hospitalizations. The evolution of the annual incidence rates of hospitalization by age were described from 14 to 21 years of age. The analyses were performed using SAS Enterprise Guide 4.3 software, SAS Institute Inc., Cary, NC and STATA 13.1 (StataCorp LP Lakeway Drive College Station, Texas 77845 USA).

### Ethical approval

A specific ethics committee approval was not required for this study. INSERM, as a health research institute, has been authorized to use the EGB database by the French data protection authority (Commission Nationale de l’Informatique et des Libertés, CNIL), provided that the researcher follows specific training with certification, as the first author (Antoine Rachas) has obtained. Then he has been authorized to access and analyze the EGB database for this study.

## Results

### Prevalence of long-term diseases at the age of 14 years

Among the 801,047 subjects included in the EGB, 61,119 were eligible and included in this analysis (S1 Fig). Among them, 3.30% (95%CI: 3.16–3.44; N = 2,019) had a LTD at the age of 14 years (Table 1) and 879 were registered for a LTD after the age of 14 years and were censored at date of LTD registration. LTDs were significantly less frequent among females (2.78%) than males (3.80%). A chronic mental health disease was present in 0.81% of subjects (0.43% of females, 1.18% of males), a non-complex chronic condition in 1.10% (0.93% of females, 1.26% of males), and a complex chronic condition in 1.39% (1.42% of females, 1.36% of males). The five most frequent diseases were persistent asthma (0.38%), autism spectrum disorders (0.27%), mental retardation (0.25%), progressive structural scoliosis (0.23%), and neurotic, emotional, and mood disorders (0.22%).

**Table 1:** Prevalence of subjects with a long-term disease at 14 years of age.

Category	Group	Conditions	All (N = 61,119)			Females (N = 29,860)			Males (N = 31,259)		
			n	Pr, %	(95%CI)	n	Pr, %	(95%CI)	n	Pr, %	(95%CI)
All	All	All	2,019	3.30	(3.16;3.44)	831	2.78	(2.59;2.97)	1,188	3.80	(3.59;4.01)
Mental	All	All	498	0.81	(0.74;0.88)	129	0.43	(0.36;0.50)	369	1.18	(1.06;1.30)
	Psychiatric diseases	Autism spectrum disorders	166	0.27	(0.23;0.31)	39	0.13	(0.09;0.17)	127	0.41	(0.34;0.48)
		Specific personality disorders	120	0.20	(0.16;0.24)	28	0.09	(0.06;0.12)	92	0.29	(0.23;0.35)
		Psychotic disorders	22	0.04	(0.02;0.06)	10	0.03	(0.01;0.05)	12	0.04	(0.02;0.06)
		Neurotic, emotional, mood disorders	135	0.22	(0.18;0.26)	37	0.12	(0.08;0.16)	98	0.31	(0.25;0.37)
		Other psychological development disorder	54	0.09	(0.07;0.11)	14	0.05	(0.02;0.08)	40	0.13	(0.09;0.17)
		Other psychiatric affections	1	0.00	(0.00;0.00)	1	0.00	(0.00;0.00)	0	0.00	(0.00;0.00)
Non-complex	All	All	671	1.10	(1.02;1.18)	278	0.93	(0.82;1.04)	393	1.26	(1.14;1.38)
	Endocrine and metabolic diseases	Type 1 and type 2 diabetes	114	0.19	(0.16;0.22)	48	0.16	(0.11;0.21)	66	0.21	(0.16;0.26)
	Respiratory diseases	Persistent asthma	235	0.38	(0.33;0.43)	88	0.29	(0.23;0.35)	147	0.47	(0.39;0.55)
		Other serious chronic respiratory insufficiency	17	0.03	(0.02;0.04)	8	0.03	(0.01;0.05)	9	0.03	(0.01;0.05)
	Vascular diseases	Chronic arteriopathies with ischemic manifestations	3	0.00	(0.00;0.00)	3	0.01	(0.00;0.02)	0	0.00	(0.00;0.00)
		Severe hypertension	3	0.00	(0.00;0.00)	1	0.00	(0.00;0.00)	2	0.01	(0.00;0.02)
	Heart disease	Severe congenital heart disease	110	0.18	(0.15;0.21)	48	0.16	(0.11;0.21)	62	0.20	(0.15;0.25)
		Other heart diseases	23	0.04	(0.02;0.06)	12	0.04	(0.02;0.06)	11	0.04	(0.02;0.06)
	Kidney diseases	Severe chronic kidney disease and primitive nephrotic syndrome	47	0.08	(0.06;0.10)	24	0.08	(0.05;0.11)	23	0.07	(0.04;0.10)
	Liver diseases	Active chronic liver disease and cirrhosis	15	0.02	(0.01;0.03)	8	0.03	(0.01;0.05)	7	0.02	(0.00;0.04)
	Inflammatory Bowel Disease	Progressive Crohn's disease and ulcerative colitis	26	0.04	(0.02;0.06)	13	0.04	(0.02;0.06)	13	0.04	(0.02;0.06)
	Malignancies	Malignant tumor, malignant disease of lymphatic tissue or blood	78	0.13	(0.10;0.16)	25	0.08	(0.05;0.11)	53	0.17	(0.12;0.22)
Complex	All	All	850	1.39	(1.30;1.48)	424	1.42	(1.29;1.55)	426	1.36	(1.23;1.49)
	Chromosome abnormalities	Down syndrome	45	0.07	(0.05;0.09)	19	0.06	(0.03;0.09)	26	0.08	(0.05;0.11)
		Other chromosome abnormalities	15	0.02	(0.01;0.03)	10	0.03	(0.01;0.05)	5	0.02	(0.00;0.04)
	Neurological and muscular diseases	Cerebral palsy	89	0.15	(0.12;0.18)	37	0.12	(0.08;0.16)	52	0.17	(0.12;0.22)
		Paraplegia	11	0.02	(0.01;0.03)	6	0.02	(0.00;0.04)	5	0.02	(0.00;0.04)
		Severe epilepsy	94	0.15	(0.12;0.18)	45	0.15	(0.11;0.19)	49	0.16	(0.12;0.20)
		Other serious neurological and muscular diseases	72	0.12	(0.09;0.15)	30	0.10	(0.06;0.14)	42	0.13	(0.09;0.17)
	Mental retardation	Mental retardation	152	0.25	(0.21;0.29)	48	0.16	(0.11;0.21)	104	0.33	(0.27;0.39)
	Immunodeficiencies	Severe primary immunodeficiency requiring prolonged treatment	9	0.01	(0.00;0.02)	1	0.00	(0.00;0.00)	8	0.03	(0.01;0.05)
		HIV infection	15	0.02	(0.01;0.03)	6	0.02	(0.00;0.04)	9	0.03	(0.01;0.05)
		Medullary deficiencies and other chronic cytopenias	3	0.00	(0.00;0.00)	1	0.00	(0.00;0.00)	2	0.01	(0.00;0.02)
	Hemolysis	Hemoglobinopathies	17	0.03	(0.02;0.04)	6	0.02	(0.00;0.04)	11	0.04	(0.02;0.06)
		Other constitutional chronic hemolysis, acquired hemolysis	5	0.01	(0.00;0.02)	3	0.01	(0.00;0.02)	2	0.01	(0.00;0.02)
	Rheumatologic and systemic diseases	Progressive rheumatoid arthritis	5	0.01	(0.00;0.02)	2	0.01	(0.00;0.02)	3	0.01	(0.00;0.02)
		Juvenile arthritis	16	0.03	(0.02;0.04)	8	0.03	(0.01;0.05)	8	0.03	(0.01;0.05)
		Progressive structural scoliosis	139	0.23	(0.19;0.27)	124	0.42	(0.35;0.49)	15	0.05	(0.03;0.07)
		Vasculitis, systemic lupus erythematosus, systemic scleroderma	3	0.00	(0.00;0.00)	2	0.01	(0.00;0.02)	1	0.00	(0.00;0.00)
	Hemorrhagic diseases	Hemophilia and serious constitutional disorders of hemostasis	13	0.02	(0.01;0.03)	3	0.01	(0.00;0.02)	10	0.03	(0.01;0.05)
		Purpura and other hemorrhagic conditions	11	0.02	(0.01;0.03)	3	0.01	(0.00;0.02)	8	0.03	(0.01;0.05)
	Endocrine and metabolic diseases	Inherited metabolic diseases requiring prolonged treatment	83	0.14	(0.11;0.17)	46	0.15	(0.11;0.19)	37	0.12	(0.08;0.16)
	Respiratory diseases	Cystic fibrosis	5	0.01	(0.00;0.02)	2	0.01	(0.00;0.02)	3	0.01	(0.00;0.02)
	Vascular diseases	Disabling stroke	21	0.03	(0.02;0.04)	11	0.04	(0.02;0.06)	10	0.03	(0.01;0.05)
	Multiple diseases	Multiple diseases	27	0.04	(0.02;0.06)	11	0.04	(0.02;0.06)	16	0.05	(0.03;0.07)

Abbreviations: 95%CI, 95% confidence interval; Pr, prevalence

### Mortality

Twenty-one subjects with a LTD and 56 with no LTD died between the ages of 14 and 21 years (Table 2). The mortality rates per 10,000 person-years were 20.9 [95%CI: 13.7–32.1] and 1.9 [1.5–2.5], respectively. Mortality rate was the highest in the subjects with a complex chronic condition (30.9/10,000 person-years [17.9–53.2]). Among those with no LTD, females were less likely to die than males, with a mortality rate ratio (MRR) of 0.2; 95%CI: 0.1–0.4. This was not the case for those with a LTD (MRR: 1.3; 95%CI: 0.5–3.4). The test for interaction between gender and LTD status was significant (p = 0.001).

**Table 2:** Overall and gender-specific mortality between 14 and 21 years of age according to long-term disease.

	All (N = 61,119)		Females (N = 29,860)		Males (N = 31,259)		MRR (95%CI), females vs. males
	n	MR (95%CI), per 10,000 PY	n	MR (95%CI), per 10,000 PY	n	MR (95%CI), per 10,000 PY	
All	77	2.6 (2.1–3.2)	19	1.3 (0.8–2.0)	58	3.8 (2.9–4.9)	0.3 (0.2–0.6)
No LTD	56	1.9 (1.5–2.5)	9	0.6 (0.3–1.2)	47	3.2 (2.4–4.2)	0.2 (0.1–0.4)
LTD	21	20.9 (13.7–32.1)	10	23.9 (12.8–44.3)	11	18.4 (10.2–33.2)	1.3 (0.5–3.4)
Mental	2	8.2 (2.1–32.9)	1	15.0 (2.1–106.6)	1	5.4 (0.8–38.6)	2.8 (0.0–217.1)
Non-complex	6	17.7 (8.0–39.5)	4	29.0 (10.9–77.4)	2	10.0 (2.5–40.1)	2.9 (0.4–32.0)
Complex	13	30.9 (17.9–53.2)	5	23.3 (9.7–55.9)	8	37.1 (18.6–74.3)	0.6 (0.2–2.2)

Abbreviations: LTD, long-term disease; MR, mortality rate; MRR, mortality rate ratio; PY, person-year; 95%CI, 95% confidence interval

### Overall hospitalizations

**Fig 1:**
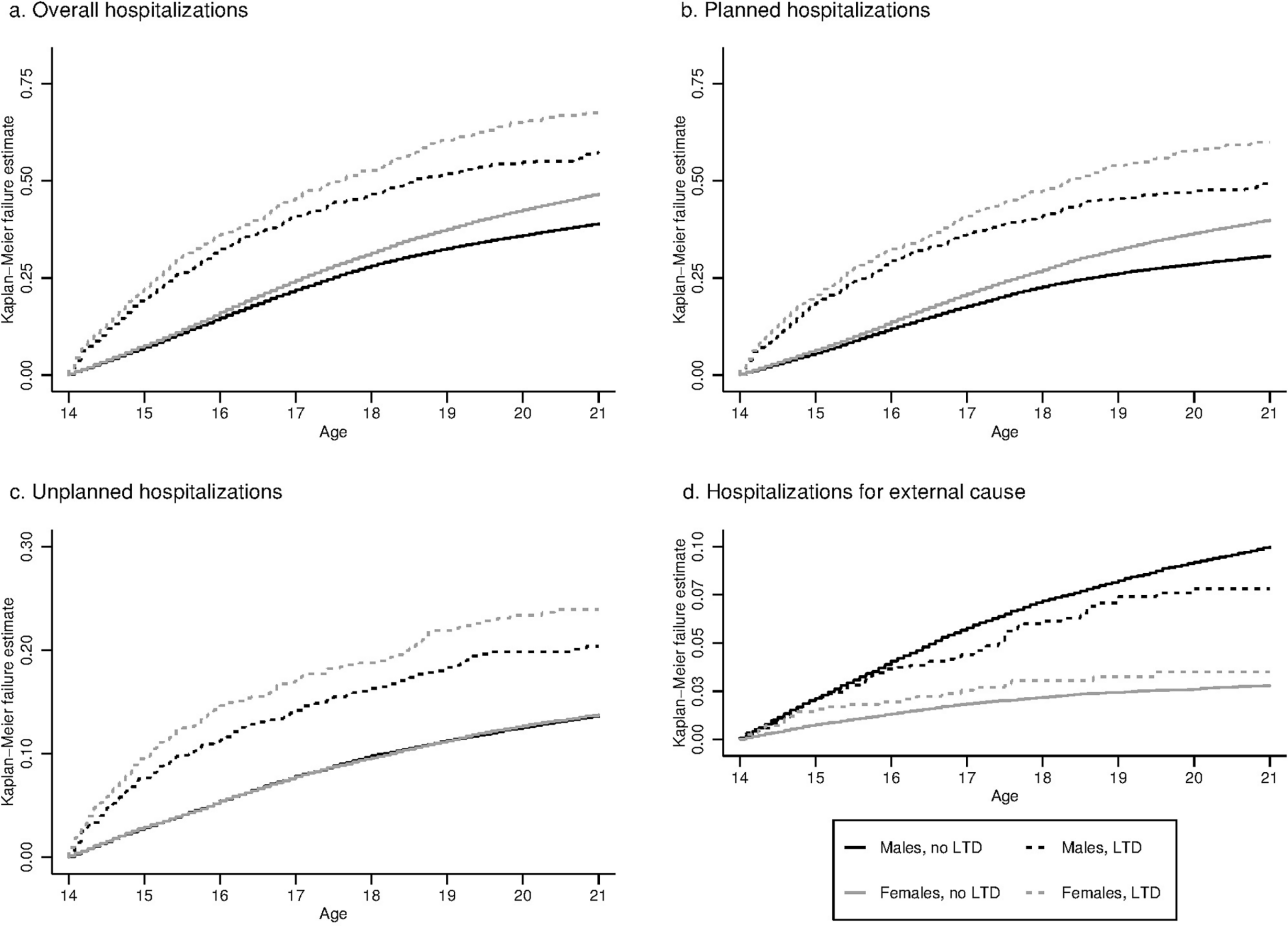
Probability of hospitalizations in short-stay units by gender and long-term disease status between the ages of 14 and 21 years (N = 61,119).

The probability of being hospitalized at least once between the ages of 14 and 21 years, estimated using the Kaplan-Meier method, was 61.8% for subjects with a LTD and 42.7% for those without (Table 3); log-rank p-value < 0.0001. The probability was the highest for subjects with a complex chronic condition (67.2%). Females were more likely to be hospitalized than males (Fig 1A), in both the LTD (67.5% vs. 57.5%, log-rank p-value < 0.001) and no LTD groups (46.6% vs. 38.9%, log-rank p-value < 0.0001).

**Table 3:** Overall and gender-specific probabilities of short-stay hospitalizations between the ages of 14 and 21 years according to the presence of a long-term disease.

Outcome	Group	All (N = 61,119)		Females (N = 29,860)		Males (N = 31,259)	
		n	Prob. (95%CI), %	n	Prob. (95%CI), %	n	Prob. (95%CI), %
≥ 1 Hospitalization	No LTD	19,629	42.7 (42.2–43.2)	10,426	46.6 (45.9–47.3)	9,203	38.9 (38.3–39.6)
	LTD	1,025	61.8 (59.1–64.5)	465	67.5 (63.5–71.4)	560	57.5 (53.9–61.0)
	Mental	192	50.7 (45.1–56.5)	65	65.6 (55.2–75.8)	127	44.8 (38.4–51.7)
	Non-complex	355	62.9 (58.4–67.3)	159	68.5 (61.6–75.1)	196	58.6 (52.9–64.5)
	Complex	478	67.2 (63.2–71.2)	241	67.4 (61.9–72.8)	237	67.0 (61.2–72.8)
≥ 1 Planned hospitalization	No LTD	16,176	35.2 (34.7–35.7)	8,883	39.9 (39.2–40.6)	7,293	30.7 (30.0–31.3)
	LTD	902	54.0 (51.3–56.7)	412	59.9 (55.9–64.0)	490	49.6 (46.1–53.1)
	Mental	155	40.9 (35.5–46.7)	53	54.4 (43.8–65.8)	102	35.8 (29.9–42.5)
	Non-complex	314	55.0 (50.6–59.6)	140	60.1 (53.2–67.2)	174	51.4 (45.7–57.3)
	Complex	433	60.7 (56.6–64.7)	219	61.4 (55.9–67.1)	214	59.7 (53.9–65.6)
≥ 1 Unplanned hospitalization	No LTD	6,338	13.7 (13.4–14.0)	3,123	13.7 (13.3–14.2)	3,215	13.6 (13.2–14.1)
	LTD	367	21.8 (19.8–24.0)	168	23.9 (20.8–27.5)	199	20.4 (17.8–23.2)
	Mental	66	17.5 (13.9–22.1)	26	27.0 (18.8–37.7)	40	14.0 (10.3–19.0)
	Non-complex	138	24.1 (20.7–28.0)	66	28.1 (22.5–34.7)	72	21.2 (17.1–26.1)
	Complex	163	22.6 (19.5–26.0)	76	20.4 (16.5–25.0)	87	24.9 (20.4–30.2)
≥ 1 Hospitalization for an external cause*	No LTD	3,016	6.4 (6.2–6.7)	663	2.8 (2.6–3.0)	2,353	10.0 (9.6–10.4)
	LTD	98	6.0 (4.9–7.3)	25	3.5 (2.4–5.2)	73	7.8 (6.2–9.8)
	Mental	25	6.7 (4.5–9.9)	5	4.4 (1.8–10.5)	20	7.5 (4.8–11.6)
	Non-complex	38	7.0 (5.1–9.6)	9	4.2 (2.1–8.1)	29	9.0 (6.3–12.9)
	Complex	35	4.9 (3.5–6.8)	11	2.8 (1.5–5.0)	24	7.0 (4.7–10.5)

n represents the number of events. Probabilities were estimated by the Kaplan-Meier method. The total number of subjects with ≥ 1 hospitalization is not the sum of numbers with planned and unplanned hospitalizations, because a subject could have both types of hospitalizations during the study period.

*different from a complication of medical or surgical care, allergy, or intoxication by non-psychotropic drugs.

Abbreviations: LTD, long-term disease; 95%CI, 95% confidence interval.

### Planned and unplanned hospitalizations

The probability of planned hospitalization by the age of 21 years was also higher for subjects with a LTD than for those without. It was also higher in females than males in both the LTD (59.9% vs. 49.6%, log-rank p-value = 0.001) and no LTD groups (39.9% vs. 30.7%, log-rank p-value <0.0001) (Fig 1B). The probability of unplanned hospitalization of subjects with a LTD was non-significantly higher for females than males (23.9% vs. 20.4%, log-rank p-value = 0.060), whereas it was almost identical between females and males without a LTD (13.7% vs. 13.6% respectively; log-rank p-value = 0.84) (Fig 1C).

The most frequent primary ICD-10 diagnosis for unplanned hospitalizations was “Injury, poisoning, and certain other consequences of external causes” for both males and females with no LTD (20.3% of stays for females, 41.6% for males) and for males with a LTD (17.5%) (S3 Table). The most frequent diagnosis for females with a LTD was “Symptoms, signs, and abnormal clinical and laboratory findings, not elsewhere classified” (16.4%). “Pregnancy, childbirth and the puerperium” represented 14.9% of the stays for females without a LTD and 4.5% for those with a LTD.

### Evolution of hospitalization rates by age

**Fig 2:**
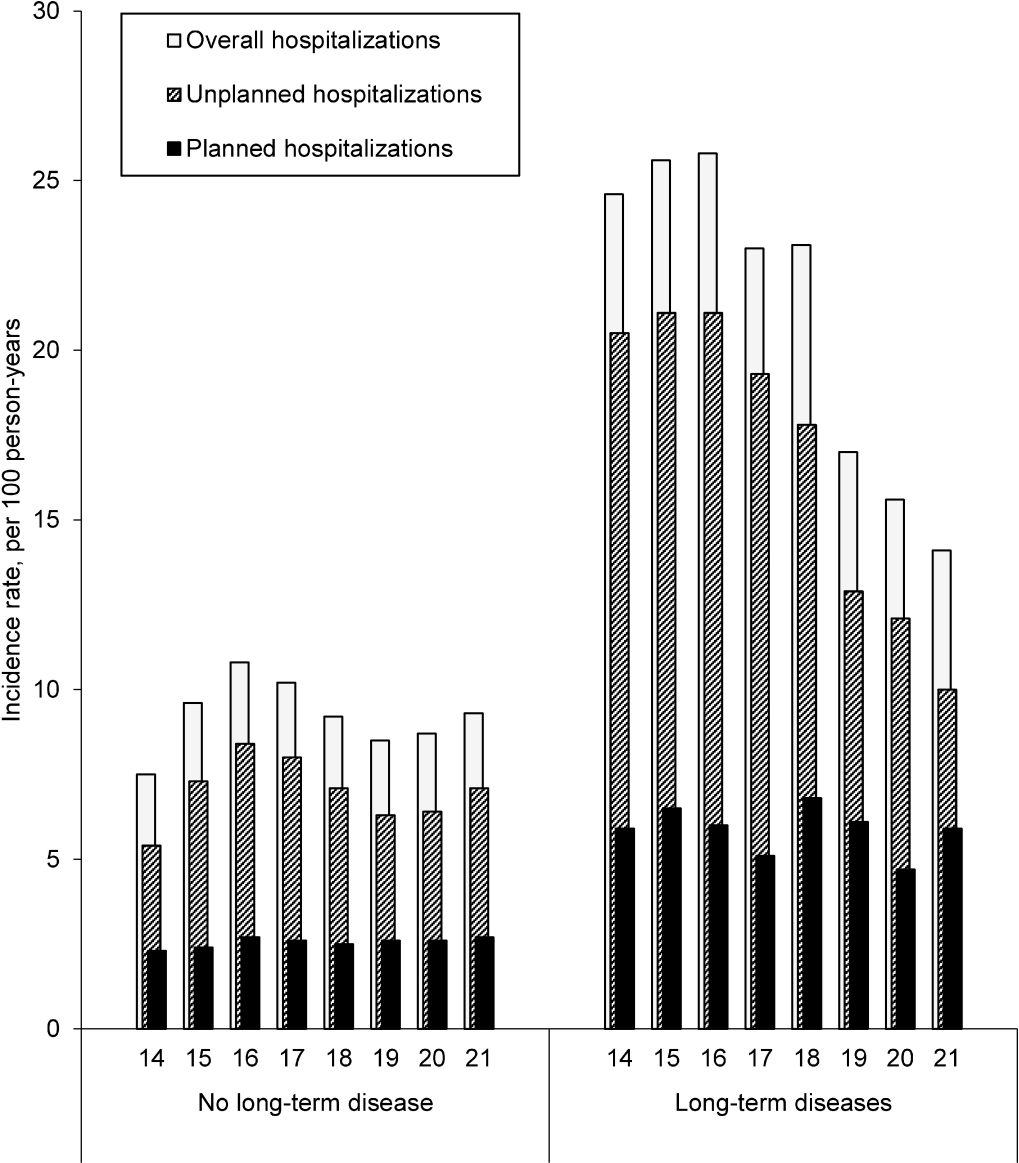
Evolution of the incidence of hospitalization in short-stay units between 14 and 21 years of age (N = 61,119).

The hospitalization rate for subjects with a LTD remained stable from 14 (24.6 per 100 person-years; 95%CI: 22.2–26.9) to 18 years of age (23.1; 20.2–26.1) and fell sharply at the age of 19 years (17.0; 14.2–19.7) to 14.1 (10.9–17.3) by the age of 21 (Fig 2). This decrease did not occur for subjects with no LTD, for which the rate of hospitalizations was 7.5 (95%CI: 7.2–7.7) at 14, 9.2 (8.9–9.5) at 18, and 9.3 (8.9–9.8) at 21 years of age, per 100 person-years. The overall decrease was the highest for youths with a complex chronic condition (S2 Fig). The fall in the rate at 19 years of age concerned planned, but not unplanned, hospitalizations, which remained stable between the ages of 14 and 21 years.

### Hospitalization for an external cause

Males in the group with no LTD were approximately 3.5-times more likely than females to be hospitalized for an external cause (10.0% vs. 2.8%; log-rank p-value < 0.0001), whereas the gender difference was smaller in the LTD group (7.8% vs. 3.5%; log-rank p-value < 0.001); p for interaction = 0.033 (Table 3, Fig 1D).

### Discussion

This study is the first to provide national estimates for the prevalence of adolescents with a LTD diagnosed before 14 years of age and their mortality and hospitalization rates based on medical data for adolescents living in France.

During the 2005–2014 period, 3.30% (95%CI: 3.16–3.44) of adolescents insured under the French general health insurance scheme had a LTD at 14 years of age. No other national studies have been published concerning the overall prevalence of youths with chronic diseases, except the 2011/2012 household National Survey of Children’s Health [23], which used a very different approach. It was based on parent-reported data and a broad definition of chronic conditions, including, for example, any kind of hearing or speech problems. In our study, the diagnoses of LTDs were double-validated by medical practitioners (the patient’s physician and the health insurance system physician). Based on this medical approach of reporting LTDs, we could focus specifically on chronic diseases that require costly and long-term care, are potentially life-threatening, or lead to handicaps or medical complications.

A strength of this study was the use of a large representative sample of the population insured under the general scheme of the French national health insurance [18], with no selection bias, as the EGB is a random sample with an anonymous procedure that does not require patient agreement. Nevertheless, the prevalence of LTDs may have been slightly underestimated for two reasons. First, some patients have not yet been diagnosed. Second, some patients are eligible for access to free care without requesting LTD registration, but this is unlikely to concern patients with the most severe conditions, who have greater healthcare needs.

A notable strength of our study was the accuracy of mortality estimates, as deaths are exhaustively registered in the French death register. The overall and gender-specific mortality rates were very close to those reported in the French national data.[24]. The mortality rates between the ages of 14 and 21 years for subjects with and without a LTD were 20.9/10,000 person-years (95%CI: 13.7–32.1) and 1.9 (1.5–2.5), respectively. The ten-fold higher mortality rate in adolescents with a LTD was expected given the severity of their diseases. However, it was still very low, even for complex chronic conditions (30.9/10000 person-years; 95%CI; 17.9–53.2). This low mortality rate in adolescents with a LTD may reflect progress in patient medical management, but also the fact that the most severe cases possibly died before the age of 14.

The higher mortality rate of males than females without a LTD is well known and was expected [24]. Mortality in young males in the general population is mostly related to external causes (e.g., trauma), supported by more frequent hospitalizations of males than females among those with no LTD for such causes in our study. This higher risk of external health issues for males reflects a behavioral gender difference in which more adolescent males partake in risky behaviors than females in the general population. Surprisingly, we did not observe such an important gender-gap among youths with a LTD and there was a significant interaction between gender and LTD for both mortality and hospitalization for an external cause. It is possible that adolescent males with a LTD are less exposed to high-risk behaviors related to serious injuries requiring hospitalizations than adolescent males of the general population because of limitations due to their disease. Further research would be needed to address this hypothesis. In addition, we cannot exclude that this result could be explained by a different distribution of more severe diseases between males and females, because detailed analysis by disease was not feasible due to the insufficient number of cases. Similarly, females might have more severe forms of the diseases, either because males with such severe forms died earlier, before 14 years, or because females might be registered for their LTD later. In the coming years, the EGB will include the cause of death, which should allow further investigation of mortality in males and females with a LTD. Stratification by gender is thus needed for any subsequent study on this topic.

Unsurprisingly, the probability of being hospitalized in a short-stay unit between the ages of 14 and 21 was higher for youths with a LTD than those without (61.8% vs. 42.7%). It was not very different or even higher for youths with a non-complex chronic condition than in those with a complex chronic condition, suggesting that non-complex chronic conditions may be very demanding and complex to manage on a daily basis. The classification used here, mainly based on single *versus* multi-organ diseases, has the advantage of indirectly grouping diseases for which the management is less well-known by specialists of adults, because they are historically pediatric multi-organ and/or rare diseases, whereas medical specialties in adult care are mostly organ-specific. Consequently, it also reflects the difficulty of pediatricians to find specialists in adult care to transfer a young adult patient. Data based on the International Classification of Functioning, Disability, and Health would be more relevant for categorizing patients according to the degree of complexity of their management, but data on activities and daily functioning are not available in the EGB.

We only included short-stay unit hospitalizations, corresponding to non-psychiatric acute health events, and hospitalizations for annual check-up for those with a chronic condition. Long-term stay units are mostly dedicated to rehabilitation and are often preceded by hospitalization in a short-stay unit (e.g., for a surgical intervention). Home-hospitalization concerns mostly dependent elderly patients. Hospitalizations in psychiatric units are not reported here, which may have led to slight underreporting of overall hospitalizations for youths with a chronic mental health condition, not hospitalized in a short-stay unit between the ages of 14 and 21 years. We estimated the one-year probability of overnight hospital stay, i.e., by the age of 15 years in the no LTD group, and found it to be 3.0% (2.8%-3.1%). This probability was quite similar than the probability estimated in a US study, which reported that 2% of 12-17-year-old US teens had experienced at least one overnight hospital stay during the previous 12 months in 2015 [25].

The hospitalization rate was higher in subjects with a LTD than in those without a LTD between the ages of 14 and 21 years and fell sharply at age 19. A decline in the hospitalization rate after 18 years of age among LTD adolescents has already been reported in a Canadian study [15]. However, the decrease for subjects over 18 years of age did not concern hospitalizations through EDs in our study and neither intensive care unit admissions or ED visits in the Canadian study. There may be several reasons for these results. First, healthcare needs may decrease with age for specific diseases, such as asthma, epilepsy, or cured cancers, but this is probably insufficient to explain the sudden fall at 19 years. Second, planned contacts with the healthcare system may decrease with changes in living conditions, as 18 is the age of legal adulthood and the end of secondary school in France. The lack of a decrease in unplanned acute care may reflect delayed regular care. According to the 2007 survey of Adult Transition and Health, adolescents (14–17 years) with special healthcare needs received significantly less timely healthcare as they aged into adulthood (19–23 years) [16]. Third, changes may be related to transfer to adult-oriented care, potentially associated with a lower frequency of medical visits [26,27], gaps in care [28], a lack of compliance with treatment regimens [29,30], or different practices between pediatricians and adult specialists (inpatient *versus* outpatient care, respectively). Scientific data on this topic are inconsistent [31–33]. We could not explore this issue, as data on the specialty of the department of hospitalization was not available in the French national health insurance database during the study period. However, access to care could be studied in a future analysis by considering data on outpatient care and consultations with private physicians, which are available in the EGB. This could help to determine whether there is a discontinuity in patient care or a transition from inpatient care to outpatient care when the patients become young adults.

## Conclusions

This study provides reference epidemiological data on youths living with a LTD in a country that aims to offer free and universal access to healthcare [34]. Approximately 3% of 14-year-old youths had a LTD. Elevated risks have been evaluated in detail for the first time in this population, in terms of mortality patterns and trends in hospitalization rates. Unexpectedly, the rate of planned hospitalization fell after age 18, the age of legal adulthood. This raises many issues, including coping strategies of young patients in high-risk situations and the role of transfer to adult care on patient behavior, prognosis, and clinical practices.

## Supporting information

S1 Fig Study flow chart. (TIF)

S2 Fig Evolution of the incidence rate of hospitalizations in short-stay units between 14 and 21 years of age, by category of long-term disease (N = 61,119). (TIF)

S1 Table Classification of long-term diseases. (DOCX)

S2 Table Repartition of the 21 deaths among youths with a long-term disease, by disease. (DOCX)

S3 Table Primary diagnosis for unplanned hospitalizations between 14 and 21 years of age. (DOCX)
