# Correction: Excess mortality and hospitalizations in transitional-age youths with a long-term disease: A national population-based cohort study

**DOI:** 10.1371/journal.pone.0195926

**Published:** 2018-04-10

**Authors:** Antoine Rachas, Philippe Tuppin, Laurence Meyer, Bruno Falissard, Albert Faye, Nizar Mahlaoui, Elise de La Rochebrochard, Marie Frank, Pierre Durieux, Josiane Warszawski

In Fig 2, the labels for “Unplanned hospitalizations” and “Planned hospitalizations” are switched. Please see the correct Fig 2 here.

**Fig 2:**
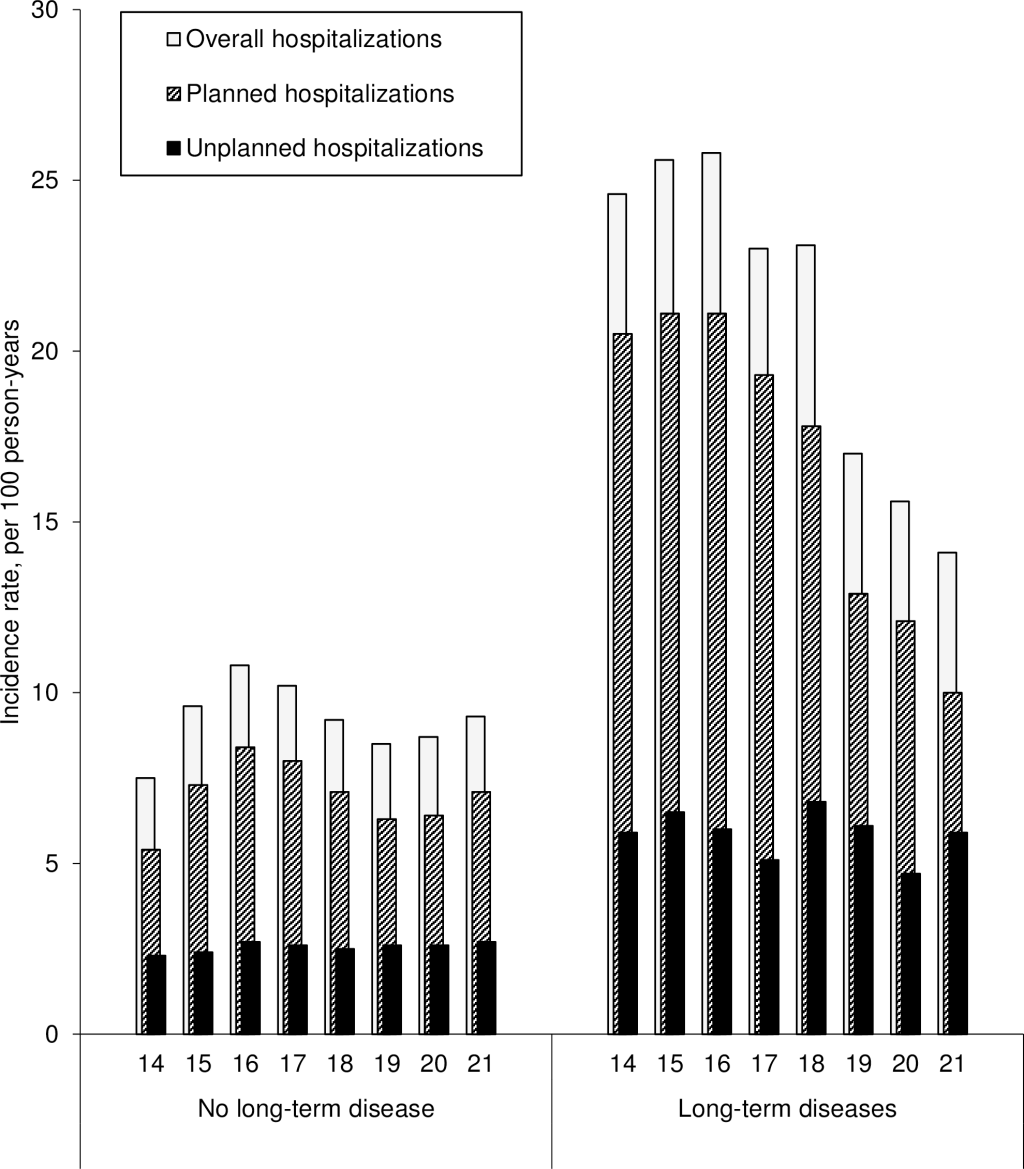
Evolution of the incidence of hospitalization in short-stay units between 14 and 21 years of age (N = 61,119).
