# Supplementary figures and images for: Excess mortality and hospitalizations in transitional-age youths with a long-term disease: A national population-based cohort study
Source: PLoS One. 2018 Mar 13;13(3):e0193729. doi: 10.1371/journal.pone.0193729 (PMC5849314; doi:10.1371/journal.pone.0193729)

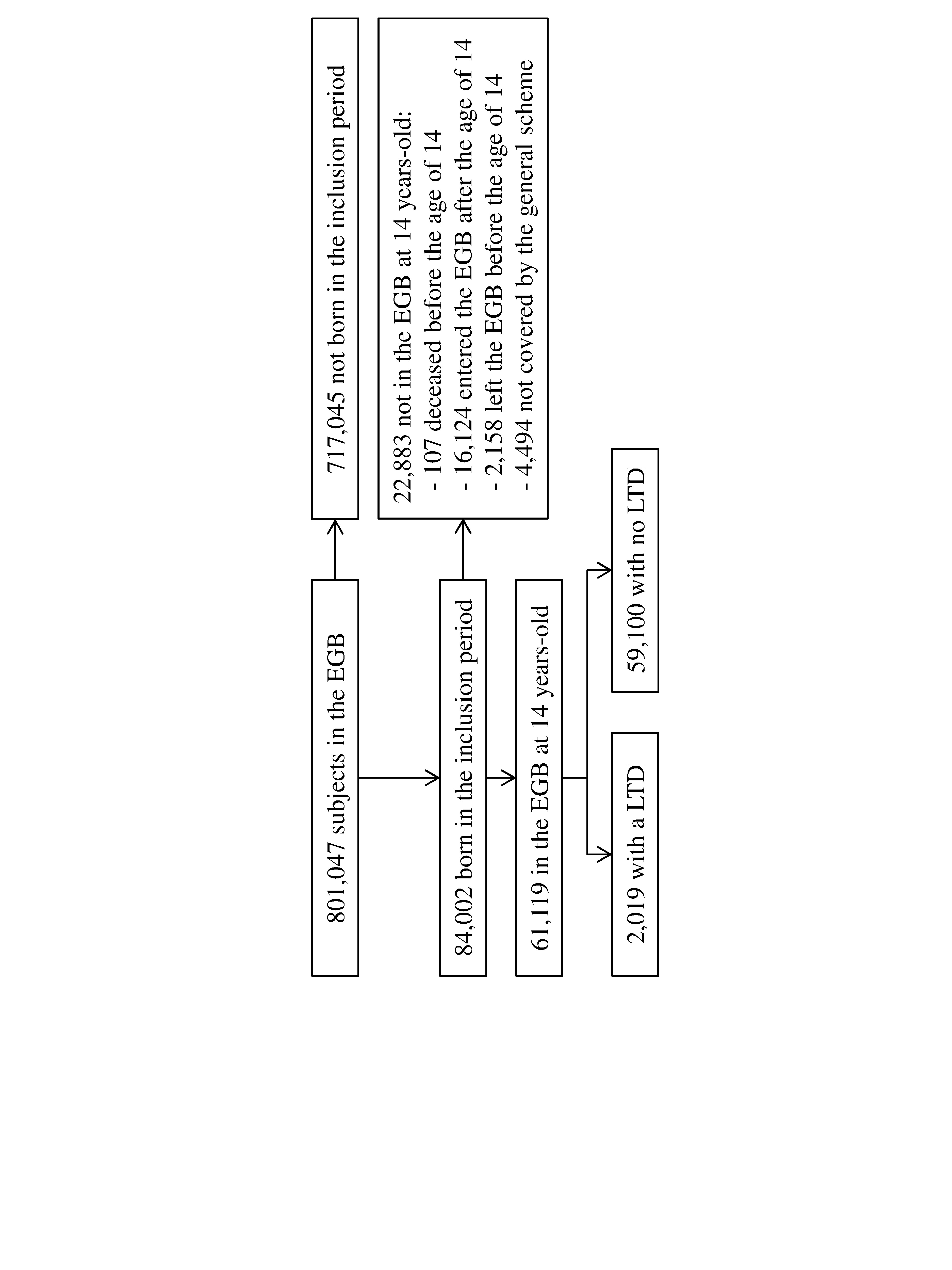

Supplement: S1 Fig — (TIF) [file pone.0193729.s001.tif]

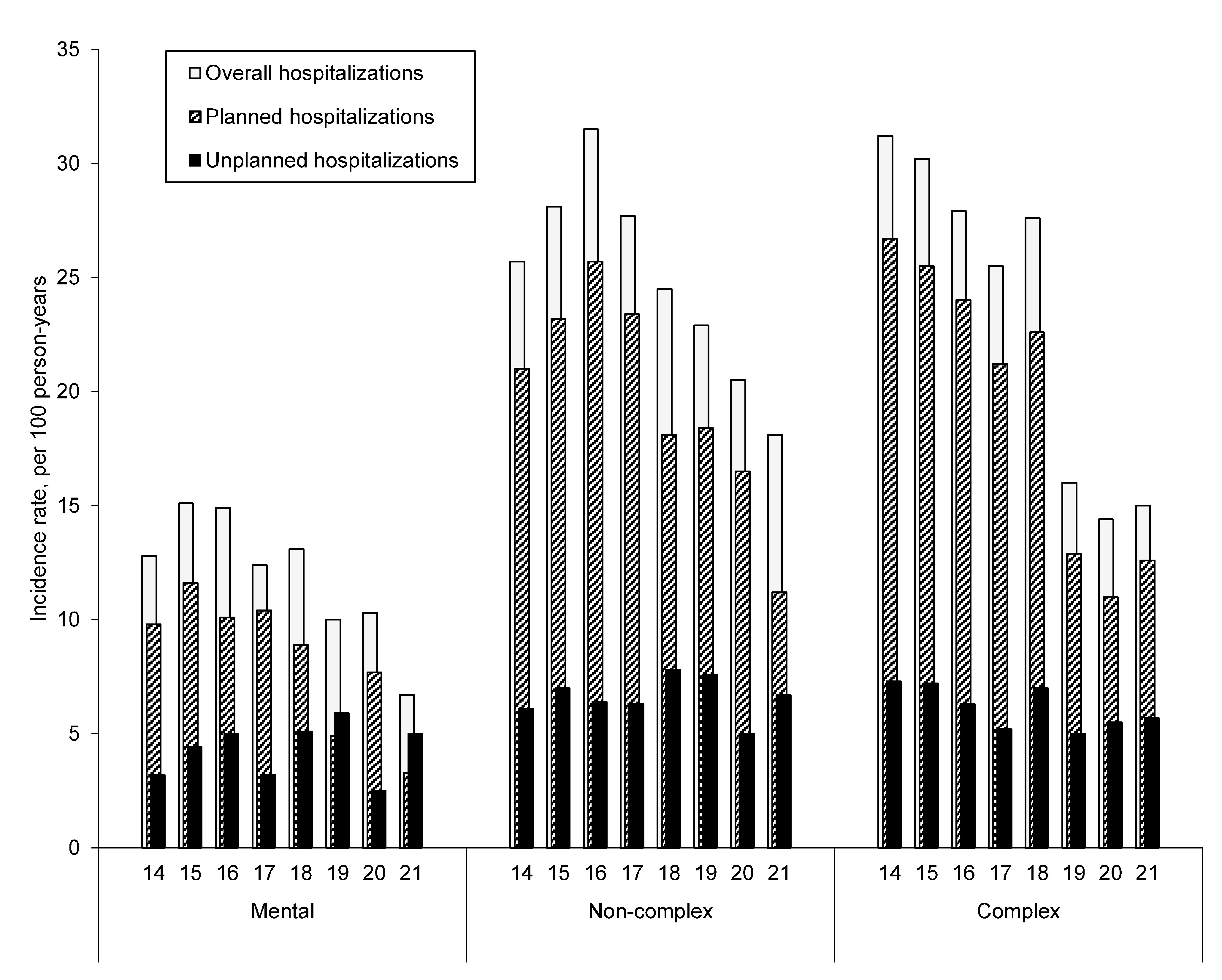

Supplement: S2 Fig — (TIF) [file pone.0193729.s002.tif]
