# Supplementary material for: Excess mortality and hospitalizations in transitional-age youths with a long-term disease: A national population-based cohort study
Source: PLoS One. 2018 Mar 13;13(3):e0193729. doi: 10.1371/journal.pone.0193729 (PMC5849314; doi:10.1371/journal.pone.0193729)
Supplement: S1 Table — (DOCX) [file pone.0193729.s003.docx]

S1 Table. Classification of long-term diseases

| Category | Group | Conditions | ICD-10 codes |
| --- | --- | --- | --- |
| Mental | Psychiatric diseases | Autism spectrum disorders | F84 |
|  |  | Specific personality disorders | F60 |
|  |  | Psychotic disorders | F20, F21, F22, F28, F29 |
|  |  | Neurotic, emotional, mood disorders | F32, F33, F38, F40, F41, F42, F43, F48, F90, F91, F92, F93, F98 |
|  |  | Other psychologic development disorder | F80, F81, F83, F88, F89 |
|  |  | Other psychiatric affections | F19, F50, F54, F59, F99 |
| Non-complex | Endocrine and metabolic diseases | Type 1 and type 2 diabetes | E10, E11 |
|  | Respiratory diseases | Persistent asthma | J45 |
|  |  | Other serious chronic respiratory insufficiency | D86, J18, J40, J41, J42, J84, J96, J98, P27, Q34 |
|  | Vascular diseases | Chronic arteriopathies with ischemic manifestations | I67, I70, I71, I73 |
|  |  | Severe hypertension | I10 |
|  | Heart diseases | Severe congenital heart disease | Q20, Q21, Q22, Q23, Q24, Q25, Q26 |
|  |  | Other heart diseases | E05, I06, I34, I35, I37, I42, I48, I49, I50, I51, P29 |
|  | Kidney diseases | Severe chronic kidney disease and primitive nephrotic syndrome | N00, N03, N04, N07, N11, N17, N18, Q60, Q62 |
|  | Liver diseases | Active chronic liver disease and cirrhosis | B18, K71, K73, K74, K76, P35, Q44 |
|  | Inflammatory Bowel Disease | Progressive Crohn's disease and Ulcerative colitis | K50, K51 |
|  | Malignancies | Malignant tumor, malignant disease of lymphatic tissue or blood | C18, C31, C38, C40, C49, C61, C68, C69, C70, C71, C72, C73, C74, C81, C83, C85, C91, C92, C94, C95, C96, D39, D42, D43, D47, D48 |
| Complex | Chromosome abnormalities | Down Syndrome | Q90 |
|  |  | Other chromosome abnormalities | Q93, Q96, Q99 |
|  | Neurological and muscular diseases | Cerebral palsy | G80 |
|  |  | Paraplegia | G82, G83 |
|  |  | Severe epilepsy | G40 |
|  |  | Other serious neurological and muscular diseases | A81, G10, G11, G12, G31, G37, G60, G61, G64, G70, G71, G72, M33, Q05, Q07, Q85 |
|  | Mental retardation | Mental retardation | F71, F78, F79, Q04 |
|  | Immuno-deficiencies | Severe primary immunodeficiency requiring prolonged treatment | D80, D81, D82, D83, D84, D89 |
|  |  | HIV infection | B20, B24, Z21 |
|  |  | Medullary deficiencies and other chronic cytopenias | D61, D70 |
|  | Hemolysis | Hemoglobinopathies | D56, D57 |
|  |  | Other Constitutional chronic hemolysis, acquired hemolysis | D58, D59 |
|  | Rheumatolo-gical and systemic diseases | Rheumatoid arthritis progressive | M05, M06, M07 |
|  |  | Juvenile arthritis | M08 |
|  |  | Progressive structural scoliosis | M41 |
|  |  | Vasculitis, systemic lupus erythematosus, systemic scleroderma | M30, M34, M35 |
|  | Hemorrhagic diseases | Hemophilia and serious constitutional disorders of hemostasis | D66, D68 |
|  |  | Purpura and other hemorrhagic conditions | D69 |
|  | Endocrine and metabolic diseases | Inherited metabolic diseases requiring prolonged treatment | E03, E07, E20, E23, E25, E70, E71, E72, E73, E74, E75, E78, E79, E83, E88, Q77, Q87 |
|  | Respiratory diseases | Cystic fibrosis | E84 |
|  | Vascular diseases | Disabling stroke | G81, I61, I62, I64, I69 |
| Abbreviations: ICD-10, international classification of diseases version 10 | | | |
