# Supplementary material for: Excess mortality and hospitalizations in transitional-age youths with a long-term disease: A national population-based cohort study
Source: PLoS One. 2018 Mar 13;13(3):e0193729. doi: 10.1371/journal.pone.0193729 (PMC5849314; doi:10.1371/journal.pone.0193729)
Supplement: S2 Table — (DOCX) [file pone.0193729.s004.docx]

S2 Table. Repartition of the 21 deaths among youths with a long-term disease, by disease

| Long-term disease | All (n=21) | Females (n=10) | Males (n=11) |
| --- | --- | --- | --- |
| Autism spectrum disorders | 1 | 1 | 0 |
| Inherited metabolic diseases requiring prolonged treatment | 1 | 1 | 0 |
| Malignant tumor, malignant disease of lymphatic tissue or blood | 4 | 2 | 2 |
| Mental retardation | 4 | 2 | 2 |
| Multiple diseases | 1 | 0 | 1 |
| Neurotic, emotional, mood disorders | 1 | 0 | 1 |
| Other chromosome abnormalities | 1 | 0 | 1 |
| Other heart diseases | 1 | 1 | 0 |
| Other serious neurological and muscular diseases | 5 | 2 | 3 |
| Severe epilepsy | 1 | 0 | 1 |
| Type 1 and type 2 diabetes | 1 | 1 | 0 |
