# Supplementary material for: Excess mortality and hospitalizations in transitional-age youths with a long-term disease: A national population-based cohort study
Source: PLoS One. 2018 Mar 13;13(3):e0193729. doi: 10.1371/journal.pone.0193729 (PMC5849314; doi:10.1371/journal.pone.0193729)
Supplement: S3 Table — (DOCX) [file pone.0193729.s005.docx]

S3 Table. Primary diagnosis for unplanned hospitalizations between 14 and 21 years of age

| Main diagnosis (ICD-10 chapter) | No LTD | | |  | LTD | | |
| --- | --- | --- | --- | --- | --- | --- | --- |
|  | All | Females | Males |  | All | Females | Males |
|  | N=8,898 stays | N=4,688 stays | N=4,210 stays |  | N=811 stays | N=422 stays | N=389 stays |
| Injury, poisoning and certain other consequences of external causes | 30.4 (2,703) | 20.3 (951) | 41.6 (1,752) |  | 14.1 (114) | 10.9 (46) | 17.5 (68) |
| Symptoms, signs and abnormal clinical and laboratory findings, not elsewhere classified | 12.4 (1102) | 14.4 (677) | 10.1 (425) |  | 14.9 (121) | 16.4 (69) | 13.4 (52) |
| Diseases of the digestive system | 10.6 (946) | 10.5 (494) | 10.7 (452) |  | 8.5 (69) | 8.5 (36) | 8.5 (33) |
| Mental and behavioral disorders | 9.2 (820) | 9.2 (431) | 9.2 (389) |  | 7.2 (58) | 9 (38) | 5.1 (20) |
| Diseases of the genitourinary system | 6.3 (561) | 8.4 (395) | 3.9 (166) |  | 2.6 (21) | 3.3 (14) | 1.8 (7) |
| Pregnancy, childbirth and the puerperium | 7.9 (699) | 14.9 (699) | 0 |  | 2.3 (19) | 4.5 (19) | 0 |
| Diseases of the respiratory system | 4 (360) | 3.2 (150) | 5 (210) |  | 5.8 (47) | 6.6 (28) | 4.9 (19) |
| Diseases of the nervous system | 3 (263) | 3.1 (143) | 2.9 (120) |  | 10.1 (82) | 9.5 (40) | 10.8 (42) |
| Certain infectious and parasitic diseases | 3 (264) | 3.1 (143) | 2.9 (121) |  | 2.7 (22) | 2.6 (11) | 2.8 (11) |
| Diseases of the skin and subcutaneous tissue | 2.8 (252) | 2.6 (123) | 3.1 (129) |  | 2 (16) | 1.2 (5) | 2.8 (11) |
| Diseases of the musculoskeletal system and connective tissue | 1.6 (143) | 1.3 (61) | 1.9 (82) |  | 2 (16) | 1.7 (7) | 2.3 (9) |
| Diseases of the circulatory system | 1.5 (132) | 1.4 (64) | 1.6 (68) |  | 1.6 (13) | 2.4 (10) | 0.8 (3) |
| Factors influencing health status and contact with health services | 1.2 (105) | 1.4 (67) | 0.9 (38) |  | 3 (24) | 3.3 (14) | 2.6 (10) |
| Endocrine, nutritional and metabolic diseases | 0.6 (57) | 0.6 (27) | 0.7 (30) |  | 10.7 (87) | 10 (42) | 11.6 (45) |
| Diseases of the blood and blood-forming organs and certain disorders involving the immune mechanism | 0.7 (63) | 0.6 (26) | 0.9 (37) |  | 7.9 (64) | 5 (21) | 11.1 (43) |
| Diseases of the ear and mastoid process | 0.3 (28) | 0.4 (21) | 0.2 (7) |  | 0.4 (3) | 0.5 (2) | 0.3 (1) |
| Diseases of the eye and adnexa | 0.3 (26) | 0.3 (16) | 0.2 (10) |  | 0 | 0 | 0 |
| Neoplasms | 0.4 (39) | 0.5 (25) | 0.3 (14) |  | 0.5 (4) | 0.2 (1) | 0.8 (3) |
| Congenital malformations, deformations and chromosomal abnormalities | 0.1 (11) | 0.1 (4) | 0.2 (7) |  | 0.5 (4) | 0.5 (2) | 0.5 (2) |
| Missing | 9.2 (820) | 9.2 (431) | 9.2 (389) |  | 7.2 (58) | 9 (38) | 5.1 (20) |
| Data are expressed as % (n). Abbreviation:ICD-10, international classification of diseases version 10; LTD, long-term disease | | | | | | | |
